# Thalamocortical Afferents Innervate the Cortical Subplate much Earlier in Development in Primate than in Rodent

**DOI:** 10.1093/cercor/bhy327

**Published:** 2019-01-21

**Authors:** Ayman Alzu’bi, Jihane Homman-Ludiye, James A Bourne, Gavin J Clowry

**Affiliations:** 1 Institute of Neuroscience, Newcastle University, Framlington Place, Newcastle upon Tyne, UK; 2 Institute of Genetic Medicine, Newcastle University, Newcastle upon Tyne, UK; 3 Department of Basic Medical Sciences, Faculty of Medicine, Yarmouk University, Irbid, Jordan; 4 Australian Regenerative Medicine Institute, Monash University, Clayton, Victoria, Australia

**Keywords:** corticothalamic afferents, human cortical development, subplate, thalamocortical afferents, thalamus

## Abstract

The current model, based on rodent data, proposes that thalamocortical afferents (TCA) innervate the subplate towards the end of cortical neurogenesis. This implies that the laminar identity of cortical neurons is specified by intrinsic instructions rather than information of thalamic origin. In order to determine whether this mechanism is conserved in the primates, we examined the growth of thalamocortical (TCA) and corticofugal afferents in early human and monkey fetal development. In the human, TCA, identified by secretagogin, calbindin, and ROBO1 immunoreactivity, were observed in the internal capsule of the ventral telencephalon as early as 7–7.5 PCW, crossing the pallial/subpallial boundary (PSB) by 8 PCW before the calretinin immunoreactive corticofugal fibers do. Furthermore, TCA were observed to be passing through the intermediate zone and innervating the presubplate of the dorsolateral cortex, and already by 10–12 PCW TCAs were occupying much of the cortex. Observations at equivalent stages in the marmoset confirmed that this pattern is conserved across primates. Therefore, our results demonstrate that in primates, TCAs innervate the cortical presubplate at earlier stages than previously demonstrated by acetylcholinesterase histochemistry, suggesting that pioneer thalamic afferents may contribute to early cortical circuitry that can participate in defining cortical neuron phenotypes.

## Introduction

It is generally accepted from studies in mice that the protomap of cortical arealisation, which determines which region of the cortex is innervated by which thalamic nucleus, is driven by intrinsic programmes of gene expression (Rakic 1988; Miyashita-Lin et al. 1999; O’Leary et al. 2007; Rakic et al. 2009; Alfano and Studer 2013) and there is evidence that this may generally hold true in primates, including humans (Šestan et al. 2001; Clowry et al. 2018). At later developmental stages, input from thalamocortical afferents (TCA) can sharpen boundaries between cortical layers, areas, and primary and higher order domains (Nakagawa and O’Leary 2003; Chou et al. 2013; Pouchelon et al. 2014). However, a recent study has found that the relative size of a cortical area is determined by waves of spontaneous activity transmitted from the thalamus to cortex via TCA from about embryonic day (E) 16 in mouse, prior to peripheral sensory inputs reaching the thalamus (Moreno-Juan et al. 2017). Therefore, understanding better the timing of thalamocortical innervation in humans will be crucial to assessing the extent to which thalamic activity drives cortical development in complex brains.

In the mouse, it is well established that TCA cross the diencephalic/telencephalic boundary (DTB) at embryonic day (E) 12, the pallial/subpallial boundary (PSB) by E14.5 and innervate the subplate by E15.5, where they make synaptic connections with subplate neurons (Miyashita-Lin et al. 1999; Auladell et al. 2000; López-Bendito and Molnár 2003; Gezelius and López-Bendito 2017; Antón-Bolaños et al. 2018). In human, in studies employing histological, principally acetylcholinesterase histochemistry, and imaging modalities (Krsnik et al. 2017; Išasegi et al. 2018) it has been reported that TCA from the ventrolateral thalamus reach the DTB boundary at 7.5 PCW, the PSB at 9.5 PCW, enter the intermediate zone (IZ) of the cortical wall by 11 PCW, innervating the presubplate (pSP) and deep cortical plate (CP) between 12 and 14 PCW, around the time these two structures fuse to form a large subplate characteristic of primates (Kostović and Rakic 1990; Wang et al. 2010; Duque et al. 2016). These fibers correspond to somatosensory thalamic afferents and are the earliest to arrive; preceding by 2 weeks thalamic innervation of anterior and posterior cortex as described in previous studies (Kostović and Goldman Rakic 1983; Kostović and Rakic 1984). In terms of cortical development, it would appear that both mice and humans follow a similar timetable, as layer VI (corticothalamic) neurogenesis begins about the age when thalamic afferents are leaving the diencephalon and they innervate the cortex at the time when layer IV (eventual target for primary thalamic afferents) neurogenesis is becoming established (http://translatingtime.org; Workman et al. 2013; Silbereis et al. 2016).

That corticothalamic neurons are born prior to the arrival of thalamic afferents is key to our current understanding of thalamocortical pathfinding to the cortex. Corticothalamic axons cross the PSB first (“handshake hypothesis”) and are required to guide the TCA across the PSB (Molnár et al. 1998; Chen et al. 2012; Molnár et al. 2012) which is dependent upon the cortically derived axon guidance molecule draxin (Shinmyo et al. 2015). Similarly, a fundamental feature of thalamocortical development is that thalamic afferents wait in the subplate for layer IV to become established in the CP before thalamic innervation of this layer takes place (Lund and Mustari 1977; Wise and Jones 1978; Kostović and Rakic 1984; Hevner 2000; Kostović and Judas 2002).

The present study examined the expression of three proteins, secretagogin (SCGN) calbindin (CalB), and ROBO1, present in both cell body and axon, in the developing human and marmoset thalamus. We were able to identify the outgrowth of axons from early born thalamic neurons and demonstrate that thalamocortical innervation occurs prior to the outgrowth of corticofugal fibers and in time to innervate the pSP from the earliest stages of CP formation.

## Methods and Materials

### Human Tissue

Human fetal tissue from terminated pregnancies was obtained from the joint MRC/Wellcome Trust-funded Human Developmental Biology Resource (HDBR, http://www.hdbr.org; Gerrelli et al. 2015). All tissue was collected with appropriate maternal consent and approval from the Newcastle and North Tyneside NHS Health Authority Joint Ethics Committee. Fetal samples ranging in age from 7 to 12 PCW were used. The stage of development was assessed on the basis of external features according to the Carnegie staging protocol (O’Rahilly and Muller 1987) from 7 to 8 PCW and from foot and heel to knee length measurements according to Hern (1984) from 8 to 12 PCW. One sample at 7 PCW, 1 at 7.5 PCW, 3 at 8 PCW, 2 at 10 PCW and 2 at 12 PCW were used.

For immunostaining, brains were isolated and fixed for at least 24 h at 4°C in 4% paraformaldehyde (Sigma-Aldrich, Poole, UK) dissolved in 0.1 M phosphate-buffered saline (PBS). Once fixed, whole or half brains (divided sagittally) were dehydrated in a series of graded ethanols before embedding in paraffin. Brain samples were cut at 8-μm section thickness in three different planes; horizontally, sagittally, and coronally, and mounted on slides.

### Marmoset Tissue

All experiments were conducted according to the Australian Code of Practice for the Care and Use of Animals for Scientific Purposes and were approved by the Monash University Animals Ethics Committee, which also monitored the welfare of the animals. New World marmoset monkeys (*Callithrix jacchus*) aged embryonic day (E) 55 (*n* = 1) and 60 (*n* = 2) were used in this study, procured from the National Nonhuman Primate Breeding and Research Facility (Australia). The animals were transcardially flushed with warm heparinised phosphate buffer 0.1 M (PB; pH 7.2) containing 0.1% sodium nitrite and subsequently perfused with 4% paraformaldehyde in PB 0.1 M. Cerebral tissues were postfixed overnight in the same fixative at 4°C, dehydrated in increasing concentrations of sucrose (10%, 20%, and 30%) in PB 0.1 M, frozen in isopropanol cooled at −49 °C and stored at −80 °C until cryosectioning.

### Histology and Immunohistochemistry

For human tissue, immunohistochemistry was carried out on paraffin sections according to previously described protocols (Harkin et al. 2016; Alzu’bi et al. 2017). Antigen retrieval involved boiling in 10 mM citrate buffer pH 6 for 10 min. Sections were incubated with primary antibody (diluted in 10% normal blocking serum in Tris buffered saline [TBS] pH 7.6) overnight at 4°C. Details of primary antibodies are found in Supplementary Table S1. Sections were incubated with biotinylated secondary antibody for 30 min at room temperature (Vector Laboratories Ltd., Peterborough, UK) 1:500 dilution in 10% normal serum in TBS followed by incubation with avidin-peroxidase for 30 min (ABC-HRP, Vector Labs) then developed with diaminobenzidine (DAB) solution (Vector Labs) washed, dehydrated and mounted using DPX (Sigma-Aldrich, Poole, UK). For double immunofluorescence, the Tyramide Signal Amplification (TSA) method was used permitting double staining using same species antibodies (Goto et al. 2015). At the secondary antibody stage, sections were incubated with HRP-conjugated secondary antibody for 30 min (ImmPRESS™ HRP IgG [Peroxidase] Polymer Detection Kit, Vector Labs) and then incubated in the dark for 10 min with fluorescein tyramide diluted at 1/500 (Tyramide Signal Amplification (TSA™)) fluorescein plus system reagent (Perkin Elmer, Buckingham, UK) leaving fluorescent tags covalently bound to the section. Sections were then boiled in 10 mM citrate buffer pH 6 to remove all antibodies and unbound fluorescein then incubated first in 10% normal serum then with the second primary antibody for 2 h at room temperature. Sections were again incubated with HRP-conjugated secondary antibody followed by CY3 tyramide for 10 min (Tyramide Signal Amplification [TSA™] CY3 plus system reagent, Perkin Elmer). Sections were dyed with 4′,6-diamidino-2-phenylindole dihydrochloride (DAPI; Thermo Fisher Scientific, Cramlington, UK) and mounted using Vectashield Hardset Mounting Medium (Vector Labs). Extensive washing of sections was carried out between all incubations.

For marmoset tissue, the whole heads were cut in the horizontal plane on a cryostat (CM3050S, Leica, Wetzlar, Germany) at a thickness of 20 μm and were collected on Superfrost Plus® microscope slides (Menzel-Gläser/Thermo Fischer) and stored at −20°C. Sections were rehydrated in PBS, blocked in a solution of PBS, 0.3% Triton-X, 10% normal goat serum and incubated with rabbit anti-calretinin (diluted 1:1000 in the blocking solution) and rabbit anti-ROBO1 (same antibody as used in the human experiments, diluted 1:200 in the blocking solution) for 16–18 h at 4°C. Sections were then washed in PBS, incubated with a goat anti-rabbit Alexa Fluor 594 secondary antibody (Molecular Probes, Invitrogen, La Jolla, CA) in the blocking solution (1:1000) for 1 h, rinsed in PBS and incubated with Hoechst (Pentahydrate bis-Benzimide, Dako) to visualize cell nuclei.

**Figure 1.**
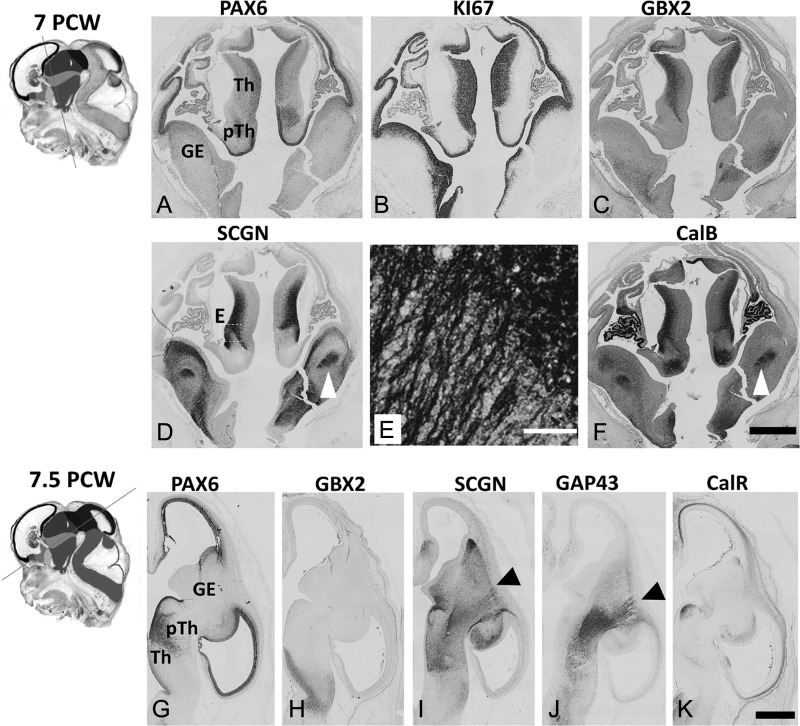
Early development of the human thalamus. Insets show plane of sectioning (see Supplementary Fig. S1 for color version and key). (*A*–*C*). At 7 PCW in the thalamus (Th) PAX6 was moderately expressed in the VZ, KI67, a marker for dividing cells, was present in both VZ and SVZ whereas GBX2 was weakly expressed in the subventricular layer but strongly expressed in an outer post-mitotic mantle layer. The prethalamus (pTh) was characterized by strong expression of PAX6 in its ventricular zone (VZ). (*D*) SCGN was expressed in both cell bodies and neurites in the outer mantle layer of the thalamus, SCGN positive fibers also seen in the internal capsule (arrowhead). E is a higher magnifcation of the boxed area in (*E*). (*F*) similarly CalB was also expressed by post-mitotic thalamic neurons and in fibers running in the IC (arrowhead). By 7.5 PCW (*G*) PAX6 expression was maintained in thalamic VZ, while in the prethalamus PAX6+ cells were now seen away from the VZ forming a boundary with the thalamus. (*H, I*) GBX2 and SCGN immunoreactivity was present in post-mitotic cells of the thalamus which extend SCGN+ positive axons to the PSB (arrowhead). (*J,L*) These axons were also GAP43 positive, but there was very little expression of SCGN, CalB or GAP43 in the cortical IZ. (*K*) however CalR was expressed in the IZ, but this expression did not reach beyond the PSB. Scale bars: 1 mm in F (and for A–D); 100 μm in E; 1 mm in K (and for G–J).

### Imaging

Images from immunoperoxidase stained sections were captured using a Leica slide scanner and Zeiss Axioplan 2 microscope; from immunofluorescent stained human sections with a Zeiss Axioimager Z2 apotome; from immunofluorescent stained marmoset sections with an Axio Imager Z1 microscope (Zeiss) equipped with a Zeiss Axiocam HRm digital camera using the Axiovision software (v 4.8.1.0) at a resolution of 1024 × 1024 pixels. The objectives used were Zeiss EC-Plan Neofluar 10×0.3, #420 340–9901. Filter sets used for visualizing fluorescently-labeled cells were Zeiss 49 Dapi #488 049-9901-000, and Zeiss HQ Texas Red #000 000-1114-462. Images were adjusted for brightness and sharpness using Adobe Photoshop CS6 software. Planes of sectioning are illustrated in Supplementary Figure S1.

## Results

### Initial Outgrowth of Human Thalamocortical Afferents (7–7.5 PCW)

At 7 PCW, the thalamic primordium was relatively underdeveloped and consisted principally of neural progenitors in a ventricular zone (VZ) around the third ventricle. These cells expressed PAX6 with moderate intensity in the thalamus (but with increased intensity in the more ventral prethalamus, Fig. 1A) as well as KI67, a marker of cell division (Fig. 1B). The lateral portion of the developing thalamus was immunopositive for the transcription factor GBX2, characteristic of developing post-mitotic thalamic projection neurons (Miyashita-Lin et al. 1999; Chen et al. 2009, Li et al. 2012; Mallika et al. 2015) and the calcium binding proteins SCGN and CalB (Fig. 1C–F), indicating the presence of a population of neurons undergoing maturation. Axons expressing SCGN and CalB were observed in the internal capsule (IC) ventral to the ganglionic eminences suggesting TCA had crossed the DTB by this stage (Fig. 1D–F).

**Figure 2.**
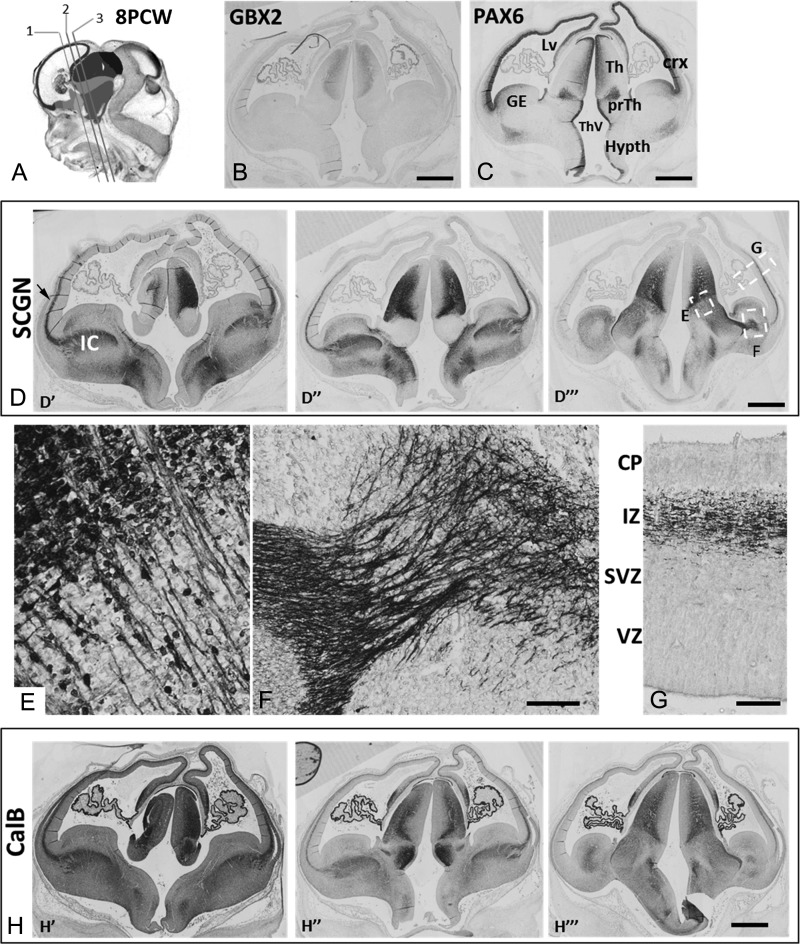
Early development of thalamocortical afferents 8 PCW. (*A*) shows planes of sectioning employed in D and H (see Supplementary Fig. S1 for color version and key). (*B*) confirms the location of post-mitotic cells of the thalamus by GBX2 immunoreactivity. (*C*) illustrates compartments of the forebrain recognized by their pattern of PAX6 immunoreactivity including the emerging cortex (crx) ganglionic eminences (GE) thalamus (Th) prethalamus (prTh) and hypothalamus (Hypth) as well as the lateral ventricle (LV) and third ventricle (ThV). (*D*) shows SCGN expression in the thalamus, and in fibers in the IC and extending into the IZ of ventrolateral cortex (arrow) at anterior (D′) intermediate (D″) and posterior (D″) levels. (*E*) SCGN was expressed in cell bodies in the thalamus and in axons traversing the DTB. (*F*) SCGN+ positive fibers were present in the IC and traversed the PSB. (*G*) SCGN+ fibers were present in the IZ. (*H*) CalB was also expressed in thalamic neurons and thalamocortical afferents that entered the cortical wall at anterior (*H*′) intermediate (*H*″) and posterior (*H*″) levels. Scale bars: 1 mm in B,C,D, 50 μm in F (and for E), 50 μm in G; 1 mm in H.

By 7.5 PCW, graded PAX6 expression remained in the thalamic VZ (Fig. 1G) and post-mitotic GBX2+ neurons were observed in the thalamus (Fig. 1H). SCGN immunoreactivity was confined to more anteroventral parts of the GBX2+ domain, whereas CalB was expressed more widely including the pretectum and prethalamus, the latter defined by expression of PAX6 in the post-mitotic cell layer (Fig. 1G, I; Supplementary Fig. S2). SCGN and CalB were expressed in the cell cytoplasm including processes, making it possible to trace axon outgrowth from the thalamus as far as the PSB from more posterior parts of the developing IC at this stage (Fig. 1I; Supplementary Fig. S2). This was confirmed by the co-expression of the growing axon marker GAP43 (Benowitz and Routtenberg 1997) in this pathway (Fig. 1J). In the cortex, at this stage, the CP is just beginning to form (Meyer et al. 2000) and no SCGN or CalB immunoreactive neurons were observed. However, calretinin (CalR) positive pioneer neurons were present in the preplate as previously described (Meyer et al. 2000).

### Thalamocortical afferents invade the human presubplate (8 PCW)

**Figure 3.**
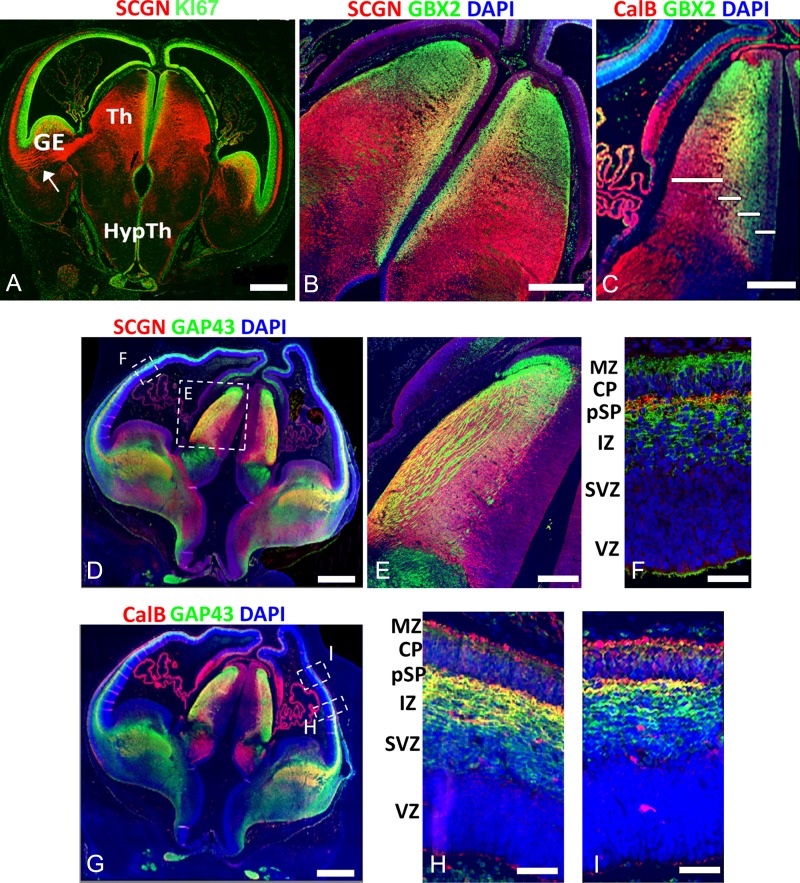
Secretagogin and calbindin expression in the human forebrain at 8 PCW. (*A*), (*B*), SCGN (red) was largely expressed in post-mitotic neurons of the thalamic mantle (M) and their axons. SCGN+ axons could be traced in continuity from the thalamus to the IZ of the ventrolateral cortical wall (arrow A). these fibers avoided PAX6+ proliferative zones of the cortical wall. B SCGN and GBX2 were co-expressed in the inner mantle of the thalamus, but GBX2 was exclusively expressed in the SVZ and more dorsal thalamic regions, whereas SCGN was confined to lateroventral thalamic regions. There was little or no expression either protein in the VZ. similarly GBX2 and CalB were co-expressed in the inner mantle, GBX2 was exclusively expressed in the SVZ and more dorsal regions, whereas CalB was confined to lateroventral regions. (*C*) The four bars denote the extent of outer mantle, inner mantle, SVZ and VZ. (*D*) SCGN+/GAP43+ axons (yellow) were observed exiting the lateral thalamus and appearing in the IC before entering the cortical wall. E shows the thalamus at higher magnification. F shows the presence of SCGN+ fibers in the presubplate (pSP) presumably of thalamic origin. GAP43+/SCGN− fibers (green) are also present in the MZ and deeper levels of the IZ of cortical or other non-thalamic origins. (*E*) CalB+ thalamic neurons also extend TCA to the pSP having first traversed the IC and IZ. (*F*) In the cortex more ventrally, SCGN+/CalB+ fibers (yellow) are present throughout the outer IZ and pSP. GAP43+/CalB− fibers (green) are present in the inner IZ and subventricular zone (SVZ). (*G*) more dorsally, SCGN+/GAP43+ thalamic afferents became confined to the pSP. CalB+ neurons and their processes (red) could be seen in the MZ and occasionally the SVZ and VZ. Scale bars: 1 mm in A,D, and G; 500 μm in B and C; 50 μm in E, F, H, and I.

By 8 PCW expression of GBX2, SCGN and CalB was evident in the post-mitotic zones of the developing thalamus; however, discrete thalamic nuclei were not formed at this stage (Fig. 2B, D, H). GBX2 and SCGN expression was restricted to the thalamus, whereas CalB was also expressed in the prethalamus, the two regions being distinguished by their patterns of PAX6 expression (Fig 1C). Co-labeling with the cell division marker KI67 showed that SCGN was expressed in the subventricular zone (SVZ) but not the VZ (Fig. 3A). GBX2 was co-expressed with SCGN and CalB in a band of cells immediately lateral to the SVZ (inner mantle, Fig. 3B, C) but was absent from the VZ. Further lateral to the inner mantle, cells and their axons predominantly expressed SCGN and CalB. Thus, the thalamus exhibited four layers, a VZ, an SVZ, an inner mantle and an outer mantle, each with a distinctive gene expression profile (Fig. 3C). The SCGN/CalB+ domain did not extend as far dorsally as the GBX2+ domain, suggesting some early arealisation of the thalamus (Fig. 3B, C).

**Figure 4.**
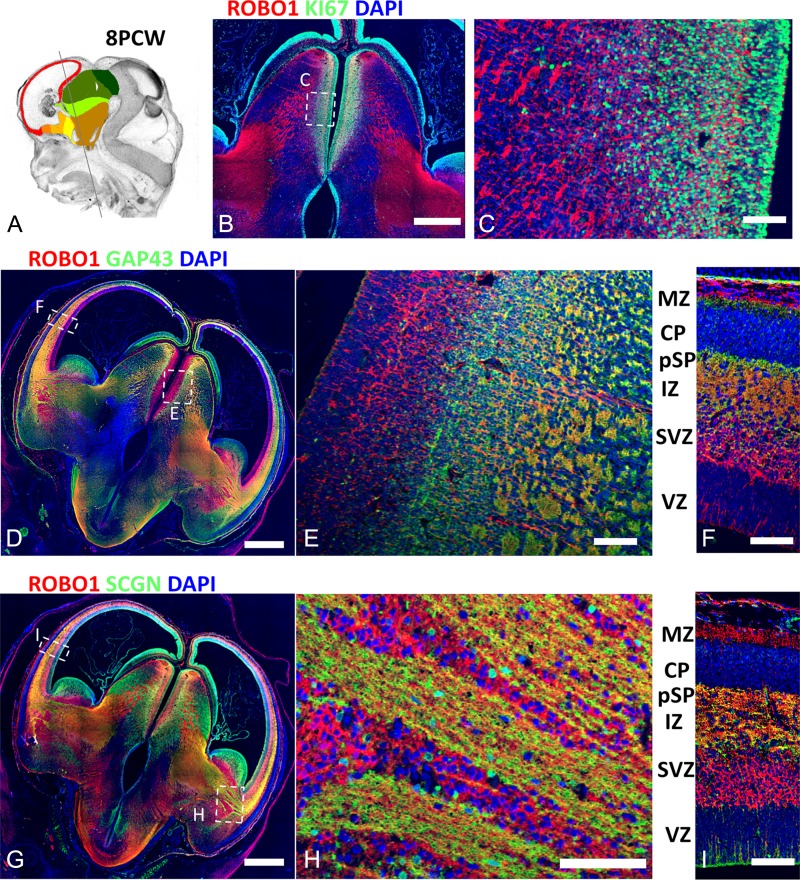
ROBO1 expression in thalamocortical afferents. (*A*) shows the plane of sectioning employed. (*B*), (*C*) confirm the expression of ROBO1in post-mitotic thalamic neurons and their afferents (red) distinct from KI67+ dividing cells (green). (*D*), (*E*), (*F*) demonstrate ROBO1+ cells of the thalamus (red) giving rise to ROBO1+/SCGN+ (yellow) axons that left the thalamus, traversed the IC and IZ to innervate the pSP. ROBO1+/GAP43− cells (red) were also observed in the cortical SVZ and MZ as previously described (Ip et al. 2011) but not in the CP. (*G*), (*H*), (*I*). ROBO1 and SCGN were co-expressed in TCA. In the IC SCGN+/ROBO1+ fibers (yellow) passed between islands of ROBO1+ cells (red). In the cortical wall, SCGN+/ROBO1+ axons were confined to the IZ and pSP. No SCGN+ or ROBO1+ neurons were present in the CP. Scale bars: Scale bars: 1 mm in B, D, and G; 50 μm in C, F, H, and I.

SCGN+ axons, emerging from SCGN+ neurons in the thalamus, extended beyond the prethalamus, into the IC and then into the IZ of the lateral cortical wall, avoiding KI67+ dividing progenitors in the cortical SVZ. (Figs 2E–G and 3A). These fiber tracts, also immunopositive for GAP43 and CalB (Fig. 3D–I), first entered the cortex at the level of the deep IZ but moved outward to reach the pSP beneath the CP (Fig. 3F, H, I). In rodent, very little GAP43 immunoreactivity is observed at the equivalent stage of development (Dani et al. 1991). GAP43+ only fibers were observed in the deep IZ and in the MZ. This is contrary to what has been described in macaque at later stages of development, where TCA run deeper in the cortical wall than corticofugal axons (Smart et al. 2002; Borello et al. 2018). No SCGN or CalB immunoreactive cells were observed in the CP or pSP (although CalB+ cells were present in the MZ) confirming that GAP43+/SCGN+ or GAP43+/CalB+ fibers in the IZ and pSP must have arisen from the thalamus.

**Figure 5.**
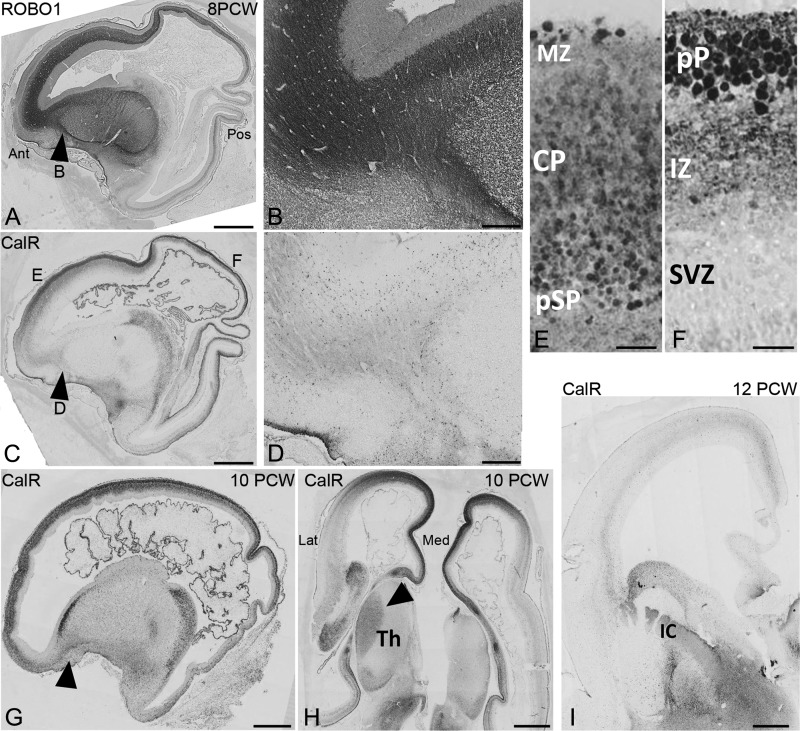
Comparison of ROBO1 and CalR expression. (*A*), A lateral saggital section at 8 PCW, shows extensive in growth of ROBO1+ axons into the frontal and parietal cortex, but less into the posterior and temporal cortex. Substantial numbers of ROBO1 immunoreactive fibers have crossed the pallial/subpalial boundary (PSB; arrowhead (*B*). In contrast (*C*) CalR+ fibers were confined to the intermediate zone (IZ) of the cortical wall but did not extend across the PSB (arrowhead, *D*). These CalR+ fibers emanate from CalR+ cell bodies localized to the Cortical plate (CP) presubplate (pSP) in anterior cortex (*E*) but in posterior cortex (*F*) where the CP has not formed yet, they arise from cells of the preplate, their axons entering the IZ. By 10 PCW (*G*) Calr+ fibers have crossed the PSB (arrowhead) and (*H*) demonstrates, in a horizontal section, CalR+ crossing the DTB (arrowhead). (*I*) weak immunoreactivity for CalR+ fibers was observed in the IZ, but expression in cell bodies was reduced. Extensive immunostaining For CalR was observed in the ventral telencephalon including the internal capsule (IC). Ant, anterior; Pos, posterior; Lat, lateral; Med, medial; MZ marginal zone; Th, thalamus. Scale Bars: 1 mm, A,C,G-I; 200 μm, B,D; 25 μm E,F.

To extend these observations further, we double labeled sections for ROBO1, a recognized marker of TCA (López-Bendito et al. 2007) with KI67, GAP43, and SCGN. ROBO1 was expressed in post-mitotic thalamic neurons, and both ROBO1+/GAP43+ and ROBO1+/SCGN+ axons were seen to extend from the lateral thalamus, through the IC and into the lateral cortex (Fig. 4). In the region of the IC, bundles of double labeled axons coursed between groups of ROBO1+/GAP43− cells (Fig. 4H). In the cortex, ROBO1+/GAP43+ and ROBO+/SCGN+ axons were confined to the upper IZ and pSP, but in addition ROBO1+/GAP43− cells were observed in the cortical SVZ (Fig. 4F, I). In sagittal sections at 8 PCW, ROBO1+ fibers were more prevalent in the anterior and parietal cortical regions compared with posterior and temporal cortex (Fig. 5A).

**Figure 6.**
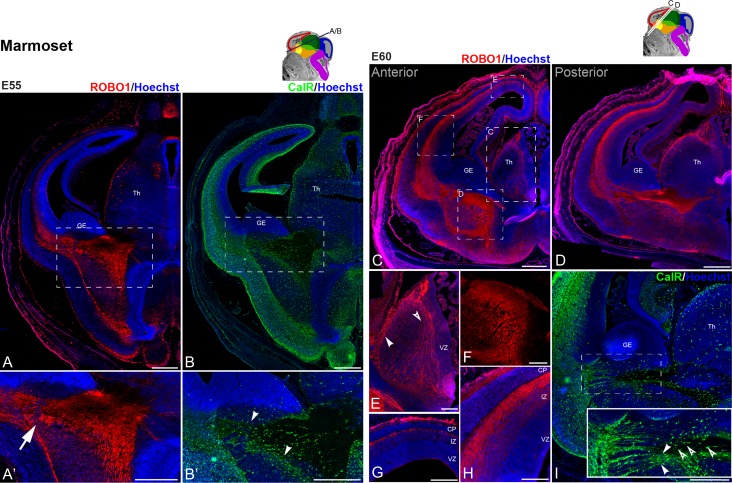
Early development of thalamocortical afferents in the marmoset. (*A*), (*B*) adjacent sections at E55 (sectioning plane illustrated on the schematic) were counterstained with a nuclear dye (Hoescht, blue, except D) to reveal the embryonic brain cytoarchitecture. ROBO1 immunoreactivity was used to label TCA (A–A′; red). ROBO1 positive fibers cell were visible extending from the thalamus into the telencephalon, invading the cortex (A′, arrow). Cortical efferents, labeled with CalR, extending from the cortex terminated in the subpallium (B-B’, arrowheads). (*C*), (*D*) nearby horizontal sections at E60 (sectioning plane illustrated on the schematic) were counterstained with a nuclear dye (Hoescht, blue, except F) and ROBO1 immunoreactivity was used to label TCA (red). ROBO1 positive cell bodies were visible in the lateral wall of the thalamus (Th) (E, arrowhead), extending thin fibers medially. Larger fascicles of ROBO1 fibers were observed in a dorso-ventral orientation (*E*, open arrowhead), running alongside the ventricular zone (VZ). These fibers merged into the IC, running ventral to the ganglionic eminence (GE) (*F*) and entered the telencephalic vesicles via the intermediate zone (IZ) of the cortical wall (*H*) targeting the presubplate (pSP, *G*) directly underneath the cortical plate (CP). While ROBO1 positive fibers dispersed in the intermediate zone (IZ), the CP and the neurogenic ventricular and subventricular (SVZ) zones were devoid of labeling. *I* Section adjacent to that illustrated in D was labeled with CalR to reveal cortical projections emerging form the cortex and entering the internal capsule (*J*, inset provides a magnified view of the PSB, arrowheads highlight CalR+ fibers entering the internal capsule, open arrowheads indicate interneurons). Scale bars: 500 μm in A–D, I; 200 μm in E–H.

### Outgrowth of Calretinin Immunoreactive Corticofugal Fibers

At 7.5 PCW CalR+ pioneer neurons were observed in the preplate (see above) and are proposed to project the first corticofugal axons (De Carlos and O’Leary 1992). Only sparse CalR and GAP43 immunoreactivity was observed in the cortical IZ (Fig. 1J, K) suggesting little axon outgrowth from cortical neurons at this age. By 8 PCW (Fig. 5C–F; Supplementary Fig. S3) CalR+ cells were still present in the preplate in posterior cortex (Fig. 5C, F) or at the boundary of the CP and pSP in more developed anterior cortex (Fig. 5C, E) and CalR+ positive axons were observed throughout the cortical IZ (Fig. 5C; Supplementary Fig. S3C, F, H). However, CalR+ fibers failed to cross the PSB into the IC (Fig. 5D; Supplementary Fig. S3B, E) whereas ROBO1+, SCGN+, or CalB+ axons were present in this location in abundance (Fig. 5B, Supplementary Fig. S3). We conclude that TCA cross the PSB boundary before CalR+ corticofugal axons.

### Early Extension of TCA in the Marmoset Neocortex

To determine if the early innervation of the pSP by TCA is unique to human development or occurs in other primates, we tested for the presence of TCA in the marmoset embryonic brain. At E55, corresponding to 7.5/8 PCW in human development, we observed ROBO1+ fibers emerging from the thalamus into the telencephalon and crossing the PSB into the cortex (Fig. 6A–A′, arrow). Comparatively, the extension of corticofugal fibers, labeled with CalR, was limited at this stage as CalR+ fibers were still contained within the cortex (Fig. 6B–B′ arrowheads). By E60, which is equivalent to 8.5/9 PCW (Homman-Ludiye and Bourne 2017) the number of ROBO1+ fibers extending across the thalamus (Fig. 6C–E) to reach the IC had substantially increased (Fig. 6C, D, F). By this stage, the ROBO1+ TCA had already crossed the PSB to invade the neocortex at the level of the IZ and the pSP (Fig. 6C, D, G, H) as we reported in the human (see above). The ROBO1+ fibers were not homogenously distributed, exhibiting a high anterior–low posterior gradient (Fig. 6G, H) as was observed in human (Fig. 5A). They also appeared more densely packed at the level of the pSP compared with the IZ (Fig. 6H). Unlike in the human, however, ROBO+ cells were not observed in the SVZ at this stage. The extension of reciprocal CalR+ presumptive corticothalamic afferents (CTA) had progressed into the IC but had not yet reached the thalamus (Fig. 6I; arrowheads) but CalR+ interneurons could be observed migrating towards the cortex (Fig. 6I, open arrowheads).

**Figure 7.**
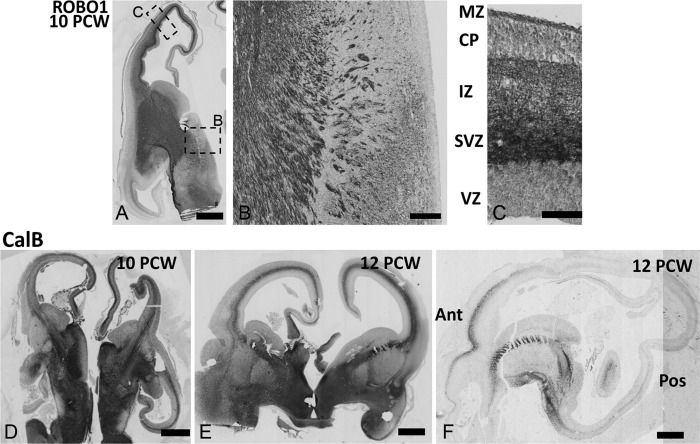
Thalamocortical afferents at 10–12 PCW. (*A*) ROBO1 expression was maintained in TCA both at the DTB (*B*) and in the intermediate zone (IZ, *C*). ROBO1 immunoreactivity intensified in the subventricular zone (SVZ) but remained weak in the cortical plate (CP). (*D, E*) At 10–12 PCW CALB immunoreactivity intensified in TCA which could be seen extending into the dorsal and medial cortex in coronal section. (*F*) In a sagittal section at 12 PCW CalB immunoreactivity was intense in the IC and in the IZ of more anterior parts of the cortex. Scale bars: 1 mm in A,D,E, and F; 200 μm in B; 50 μm in C.

### TCA Extend Further into the Human Cortex Between 8 and 12 PCW

By 10 PCW, expression of SCGN was downregulated in the thalamus, but maintained in the epithalamus (Supplementary Fig. S2B). ROBO1 and CalB expression was retained and TCA that were positive for these markers extended further dorsally, medially, posteriorly and anteriorly in the cortical wall (Supplementary Fig. S2B, Fig. 7D). ROBO1 immunoreactivity strengthened in the cortical SVZ but was still present in the IZ and pSP (Fig. 7A–C). At 12 PCW, CalB+ positive fibers were present in both medial and lateral IZ and pSP (Fig. 7E) but immunoreactivity was stronger in anterior than posterior cortex (Fig. 7F).

## Discussion

The present study demonstrates that, in the human forebrain by 7 PCW, a population of thalamic neurons is born that extends axons towards the cortex, reaching the PSB a few days later. The pSP is innervated by 8 PCW at the earliest stages of CP formation and prior to extensive outgrowth of corticofugal fibers. This was also shown to be the case in marmoset at a similarly early stage of development. It differs markedly from observations made in rodents which suggest that thalamic and cortical neurons are generated synchronously and also extend axons at the same. time This is also considerably earlier than has been previously reported from studies in human that relied principally on AChE histochemistry to visualize thalamic neurons and their afferents. This may be because AChE is not expressed by all developing TCA (Kostović and Rakic 1984). TCA are postulated to have roles in guiding cortical development. In primate development, this may happen over a more extended period and from an earlier starting point.

### Origin of the Human Thalamus

The developmental origin of the thalamus is in prosomere 2 of the diencephalon (Puelles and Rubenstein 1993; Chatterjee and Li 2012). In rodents, studies have shown that the location of thalamic neurons projecting to the cortex, as opposed to habenula and prethalamic regions within p2, is characterized by expression of the transcription factor *Gbx2* in post-mitotic cells along its anterior–posterior axis (Bulfone et al. 1993; Chen et al. 2009). Production of *Gbx2+* neurons begins as early as E10.5 and continues until E15 (Antón-Bolaños et al. 2018). All thalamic neurons express *Gbx2* at some point in their development, although expression is downregulated early in some thalamic nuclei, and persists into maturity in others (Jones & Rubenstein 2004; Chen et al. 2009; Li et al. 2012). In all cases GBX2 is essential for axon outgrowth and pathfinding (Miyashita-Lin et al. 1999; Chatterjee et al. 2012) and is required to suppress expression of markers of habenular identity (Chen et al. 2009: Mallika et al. 2015).

**Figure 8.**
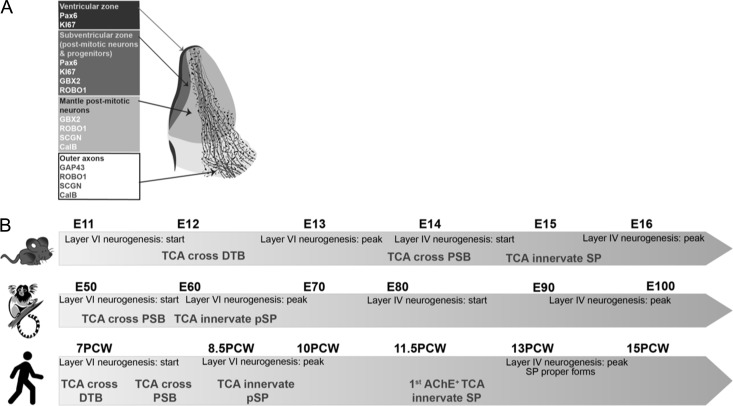
Summary diagram. *A* Localization of protein expression observed in compartments of the human thalamus during early development (7–8 PCW). *B* comparison of timelimes of development of TCA and neocortex in mouse, marmoset and human.

Similarly, in human diencephalon, we identified a region of GBX2 immunoreactivity in the post-mitotic mantle at 7 PCW, equivalent to E11 in the mouse. The number of cells in this region grew progressively over the ages observed in this study, but there was no clear evidence for the formation of discrete thalamic nuclei by 12 PCW, equivalent to E14.5 in mouse. Expression of three other proteins was co-localized with GBX2; SCGN, CalB and ROBO1. As these proteins were localized to the cytoplasm or cell membranes, revealing cell bodies and neurites, we were able to trace the course of growing TCA. A summary of the patterns of immunoreactivity we observed in the developing human thalamus is presented in Figure 8A.

### Early Arrival of TCA in the Primate Cerebral Cortex

We have demonstrated that as early as 7 PCW in human, TCA have traversed the DTB and reached the PSB by 7.5 PCW. In mouse, crossing from the DTB to the PSB takes around 2 days, and it would appear in human it only takes two or three days longer. However, rates of development of the human dorsal telencephalon are considerably slower than in mouse. Whereas Layer VI in mouse neocortex is generated within 2 days, it takes at least 18 days for this to occur in human, beginning around 7 PCW (http://translatingtime.org; Workman et al. 2013). Even if corticothalamic neurons start extending axons while still migrating to the CP, TCA reach the PSB before a substantial majority of corticothalamic neurons are even born, let alone extend axons across the PSB. Even potential CTA from CalR+ preplate pioneer neurons do not appear to project beyond the PSB by 8 PCW. Similarly, in marmoset brains, TCA also crossed the PSB before corticofugal axons left the cortex. A comparison between species is summarized in Figure 8B.

Therefore, we must conclude that the “handshake” which is required for CTA to guide TCA across the PSB in rodents may occur at a different meeting point in both marmoset and human brains, perhaps having a different role such as guiding TCA to different regions of the cortex. Our data shows that early in development TCA and CTA segregate into two different compartments within the IZ of the cortical wall, the TCA preferring to be close to the CP, whereas corticofugal axons (GAP43+ and sometimes CalR+) cluster nearer to the SVZ. Similarly, in the IC by 12 PCW, Putative CalR+ coritcofugal fibers segregate dorsally (nearer the SVZ) and TCA ventrally.

### Early Innervation of the Presubplate

Previous studies in macaque have shown that by the time of neurogenesis of the upper layers of the visual cortex, TCA lie close to the SVZ (Smart et al. 2002) and it has been proposed that they influence the rates of neurogenesis creating the difference in neuron numbers seen in areas 17 (V1) and 18 (V2) of the visual cortex (Borello et al. 2018). However, this is based on using AChE histochemistry as a marker for TCA, which does not mark all TCA (Kostović and Rakic 1984). At the stages studied here, in both human and marmoset, TCA take a more superficial route targeting the pSP which, along with the MZ, are the first laminae of the cortical wall where synapse formation takes place. This happens as early as 8–10 PCW in human, as has been shown by both ultrastructural studies (Kostović and Rakic 1990) and by immunohistochemistry for synaptophysin, neurexin 2α and vesicular GABA transporter (Bayatti et al. 2008; Harkin et al. 2017).

It has been proposed that GABAergic and glutamatergic subplate neurons form synaptic and gap junctional networks that generate oscillatory activity that is transmitted to less mature cortical neurons via gap junctions containing connexin 36 (Dupont et al. 2006; Luhmann et al. 2009, 2016). These networks may be driven or modulated by thalamic and other inputs (Luhmann et al. 2009). However, this research largely derives from studies in neonatal rodents, although it is known that low frequency tetrodotoxin sensitive calcium transients are present as early as E16 (Corlew et al. 2004) and that some neurons of the pSP and MZ are already capable of firing reliable action potentials and receiving glutamatergic and GABAergic inputs (Picken Bahrey and Moody 2003; Kilb et al. 2011). Nevertheless, E16 in mouse is still a later stage of development than 8 PCW in human, being equivalent to 15 PCW (http://translatingtime.org; Workman et al. 2013; Fig. 8**B**). No studies of electrical activity in human subplate have been made before 16 PCW, although at this age there are subplate neurons present capable of repetitive firing of action potentials (Moore et al. 2009).

Here, we are proposing that pSP driven networks may be active as early as 8 PCW in human and E60 in marmoset (Fig. 8**B**). Certainly, there is expression of human *GJDR2* mRNA, the gene for connexin 36, at 7.5–9 PCW in samples from the whole cortical wall (Lindsay et al. 2016). Expression levels (normalized RPKM 9.73 ± 3.37 SEM) are in the third quintile compared with expression of all protein coding genes between 7.5 and 17 PCW in the human cerebral cortex (Harkin et al. 2017). Evidence presented in the present study suggests TCA may also be in a position to influence this network at this stage, perhaps alongside other ascending inputs such as the sparse catecholaminergic innervation present at this time (Zecevic and Verney 1995). Thus, spontaneous activity in thalamic neurons, transmitted via the pSP to the CP, could influence expression of genes governing cortical arealisation in human cortical neurons as has been described in mouse from E16 onwards (Moreno-Juan et al. 2017) but at a much earlier stage of development. Perhaps the greater complexity of cortical arealisation in human and monkey (Buckner and Krienen 2013; Mundinando et al. 2015; Clowry et al. 2018; Krubitzer and Prescott 2018) requires that this process begins at an earlier stage. Further *in vitro* electrophysiological and molecular neuroanatomical studies are required to test this idea.

### Differential Expression of SCGN and CalB

Although both these EF-hand calcium binding proteins (and CalR) are similar in sequence and structure, they may have different functions which would explain their differing expression patterns. SCGN has a very high affinity for calcium and acts as a calcium sensor (Rogstam et al. 2007; Khandewal et al. 2017) whereas CalB has a moderate to high affinity for calcium and could act both as a sensor and calcium buffer (Schwaller 2009). SCGN is known to be important to exocytosis in certain cells, for instance from pancreatic beta cells and neuroendocrine cells of the hypothalamus (Wagner et al. 2000; Romanov et al. 2015; Yang et al. 2016). Exocytosis is the mechanism by which new cell membrane is added to the growth cone (Tsaneva-Atanasova et al. 2009; Zylbersztejn and Galli 2011) involving the synapse related SNARE (soluble *N*-ethylmaleimide-sensitive fusion attachment protein receptor) proteins (Kunwar et al. 2011). SCGN interacts with the SNARE protein SNAP25 in response to binding calcium (Rogstam et al. 2007) and could thus play a role in regulating neurite outgrowth, although it should be noted that thalamic axons grow normally in SNAP25^−/^^−^ mice (Molnár et al. 2002). Thalamic neurons are spontaneously active during the phase of axon extension (Moreno-Juan et al. 2017) and calcium transients might stimulate this process. In addition, activity, and cytosolic calcium, is shown to increase as the target is reached (Moreno-Juan et al. 2017) signaling the axon to stop and make synapses. At this stage of development, CalB is required for both calcium sensing and buffering.

## Conclusion

Up until now, in both primate and rodent, all evidence has pointed towards TCA entering the cortex after CTA have left, being guided by the CTA in the process. Our evidence shows that at least a population of pioneer TCA are likely to cross the PSB before any corticofugal afferents do. Furthermore, some TCA contact the pSP by 8 PCW in the human, at least one month earlier than previously described. This creates the possibility for thalamic input to influence subplate driven early cortical network activity from a much earlier developmental stage.

## Supplementary Material

Supplementary Data
