# Corrigendum: Thalamocortical Afferents Innervate the Cortical Subplate much Earlier in Development in Primate than in Rodent

**DOI:** 10.1093/cercor/bhz056

**Published:** 2019-04-30

**Authors:** Ayman Alzu’bi, Jihane Homman-Ludiye, James A Bourne, Gavin J Clowry

**Affiliations:** 1 Institute of Neuroscience, Newcastle University, Framlington Place, Newcastle upon Tyne NE2 4HH, UK; 2 Institute of Genetic Medicine, Newcastle University, Newcastle upon Tyne NE1 3BZ, UK; 3 Department of Basic Medical Sciences, Faculty of Medicine, Yarmouk University, Irbid, 11263, Jordan; 4 Australian Regenerative Medicine Institute, Monash University, Clayton, Victoria 3800, Australia

*Cerebral Cortex*, bhy327, https://doi.org/10.1093/cercor/bhy327

Due to confusion, the captions and figures were published originally out of order. The caption for Figure one was with Figure 2, the caption for Figure two was with Figure 3, the caption for Figure three was with Figure 4, and so on. All of the captions for each figure have since been switched into their correct order. The publisher apologizes for this error.

