# Supplementary material for: Thalamocortical Afferents Innervate the Cortical Subplate much Earlier in Development in Primate than in Rodent
Source: Cereb Cortex. 2019 Jan 21;29(4):1706–18. doi: 10.1093/cercor/bhy327 (PMC6418397; doi:10.1093/cercor/bhy327)
Supplement: Supplementary Data [file bhy327_supplementary_materials.zip › bhy327_Supplementary_materials.docx]

**Supplementary Table 1: Primary antibodies used in this study**

| **Primary antibody** | **Species** | **Dilution** | **Supplier** | **RRID or**  ***Catalogue number*** |
| --- | --- | --- | --- | --- |
| PAX6 | Rabbit polyclonal | 1/1500 | Cambridge Bioscience, Cambridge, UK. | AB_2565003 |
| GBX2 | Rabbit polyclonal | 1/500 | Proteintech, Manchester, UK. | *21639-1-AP* |
| KI67 | Mouse monoclonal | 1/150 | Dako, Ely, UK. | AB_2142378 |
| SCGN | Rabbit polyclonal | 1/500 | Sigma-Aldrich, Poole, UK. | AB_1079874 |
| CalB | Rabbit polyclonal | 1/1000 | Swant, Marly, Switzerland. | AB_10000340 |
| CalR | Mouse monoclonal | 1/500 | Merk Millipore, Watford, UK. | AB_94259 |
| CalR | Rabbit polyclonal | 1/1000 | Swant, Marly  Switzerland | AB_2721226 |
| GAP43 | Mouse monoclonal | 1/200 | Santa Cruz, Heidelberg, Germany. | AB_627660 |
| ROBO1 | Rabbit polyclonal | 1/1500 | Abcam, Cambridge, UK | AB_449561 |

**Supplementary Figure legends**

**Suppl Figure 1**

**Schematic representations of the sectioning planes employed in the study.**  A and B are expanded versions of the insets in Figure 1, C an expanded version of Figure 2A, and D an expanded version of the inset in Figure 5. Sectioning lines are marked on coronal sections adapted from optical tomograhy images (human; [www.HDBR.org](http://www.HDBR.org) ) or MRI scans (marmoset). Regions of the developing brain are filled in and colour coded.

**Suppl Figure 2**

**Double labelling studies of anti secretagogin and calbidin immunoreactivity in horizontal sections. A**  at 7.5 PCW SCGN immunoreactivity was widespread in the thalamus (Th) prethalamus (pTH) septum (Sep) internal capsule (IC) and caudal ganglionic eminence (CGE) but there was no expression in the hypothalamus (Hpth). A sharp expression boundary for SCGN between thalamus and hypothalamus is marked by asterisks. Calbindin (CalB) was expressed in all the same locations as SCGN but also in the hypothalamus and preplate /marginal zone of the dorsal telencephalon (DT). SCGN+/CalB+ fibres coursed through the IC up to the PSB (arrow). **B** at 10 PCW, SCGN expression was downregulated in the thalamus and became confined to the epithalamus (Ep) CGE and lateral ganglionic eminence (LGE). CalB expression was maintained and CalB+ TCA were present throughout the IZ of the dorsal telencephalon.

Scale bars: 1 mm in A and B.

**Suppl Figure 3**

**Double labelling studies of calretinin with ROBO1, secretagogin and calbindin.**

In an 8PCW coronal section (**A-C**) strong ROBO1 immunoreactivity (green) was seen extensively throughout the thalamus, internal capsule (IC) and lateral cortical intermediate zone (IZ) and presubplate (pSP) whereas CalR immunoreactivity (red) was present in neuronal cell bodies of the cortical plate (CP) and prethalamus (pTh) but weakly expressed in the IZ and not present in the IC or thalamus. **D-F** illustrate CalR (red) SCGN (green co-labelling at 8PCW and further emphasise the lack of CalR immunoreactive fibres crossing the PSB to the ventral telencephalon at this stage (**E**). Similarly for CalR (red) and Calb (green) co-labelling (**G, H**). Presumptive CalB+ TCA invaded the pSP but CalR+ fibres were not evident in the IC or thalamus. However by 12 PCW (**I**) both CalR and CalB are evident in the IC, with CalR+ fibres tending towards dorsal locations, and CalB+ fibres localising more ventrally.

Scale bars: I mm in A, D, G; 200 µm in B; 100 µm in C, E, F, H, I.
