# Supplementary figures and images for: Thalamocortical Afferents Innervate the Cortical Subplate much Earlier in Development in Primate than in Rodent
Source: Cereb Cortex. 2019 Jan 21;29(4):1706–18. doi: 10.1093/cercor/bhy327 (PMC6418397; doi:10.1093/cercor/bhy327)

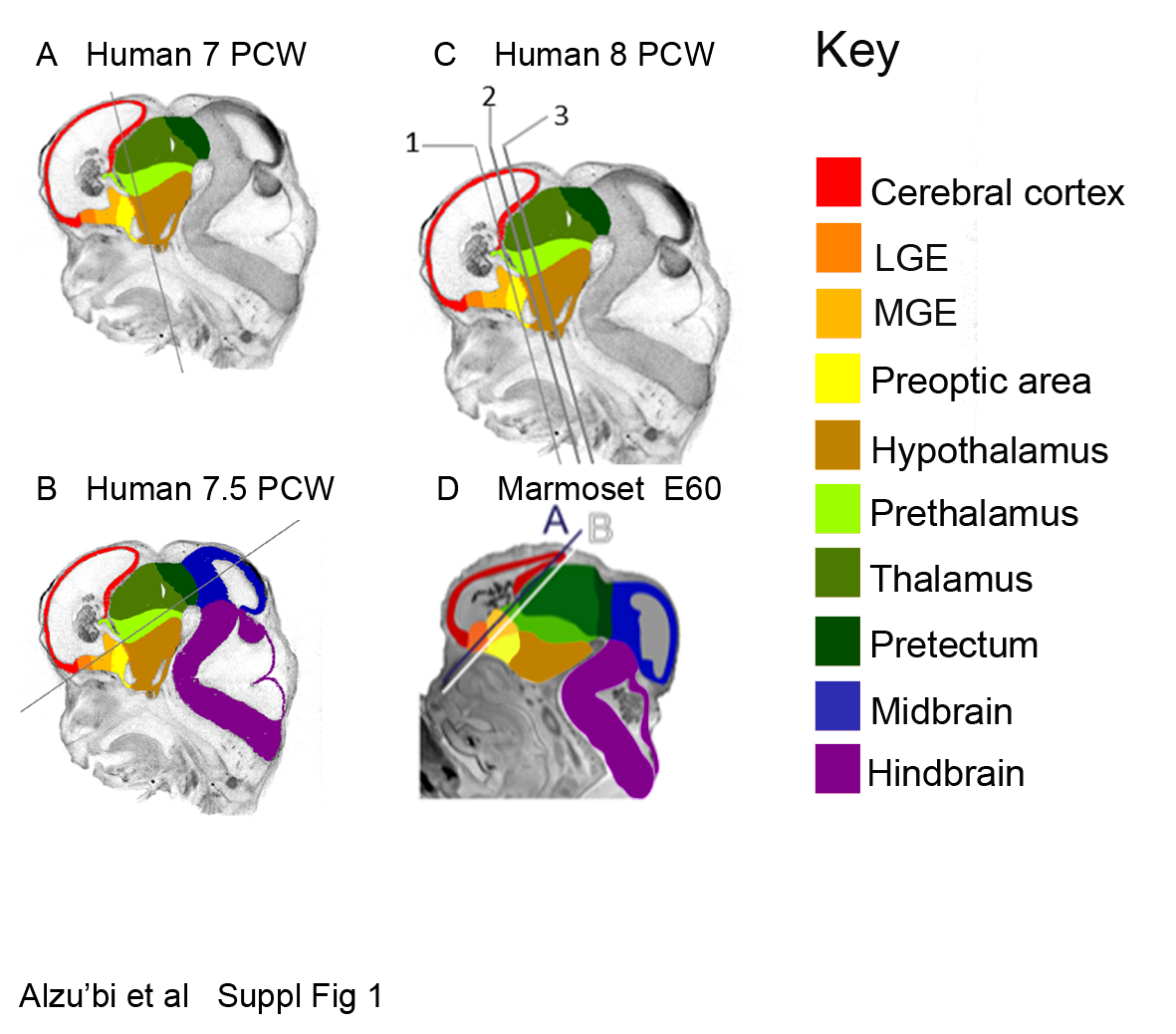

Supplement: Supplementary Data [file bhy327_supplementary_materials.zip › bhy327_Suppl_Fig_1.tif]

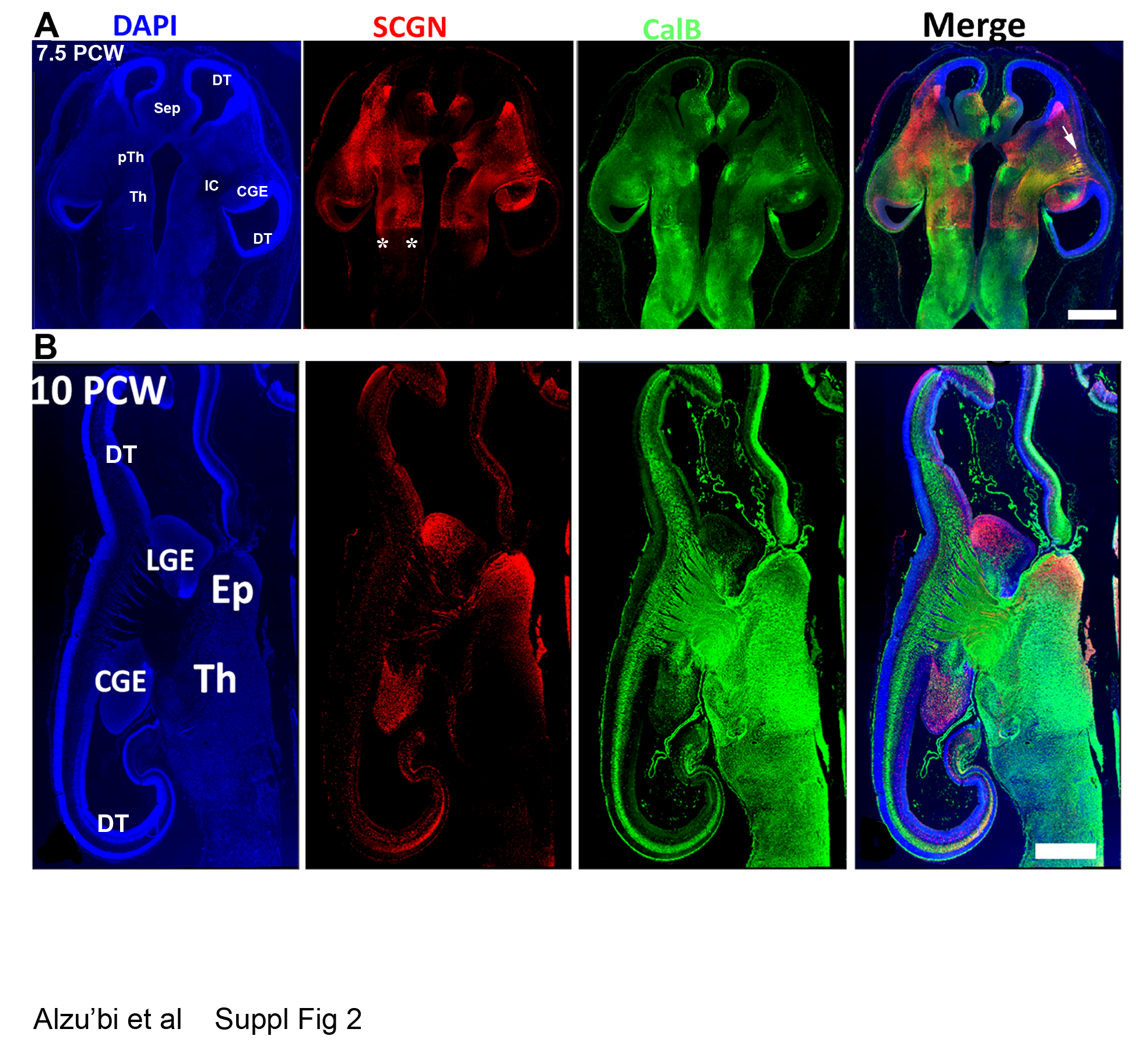

Supplement: Supplementary Data [file bhy327_supplementary_materials.zip › bhy327_Suppl_fig_2.tif]

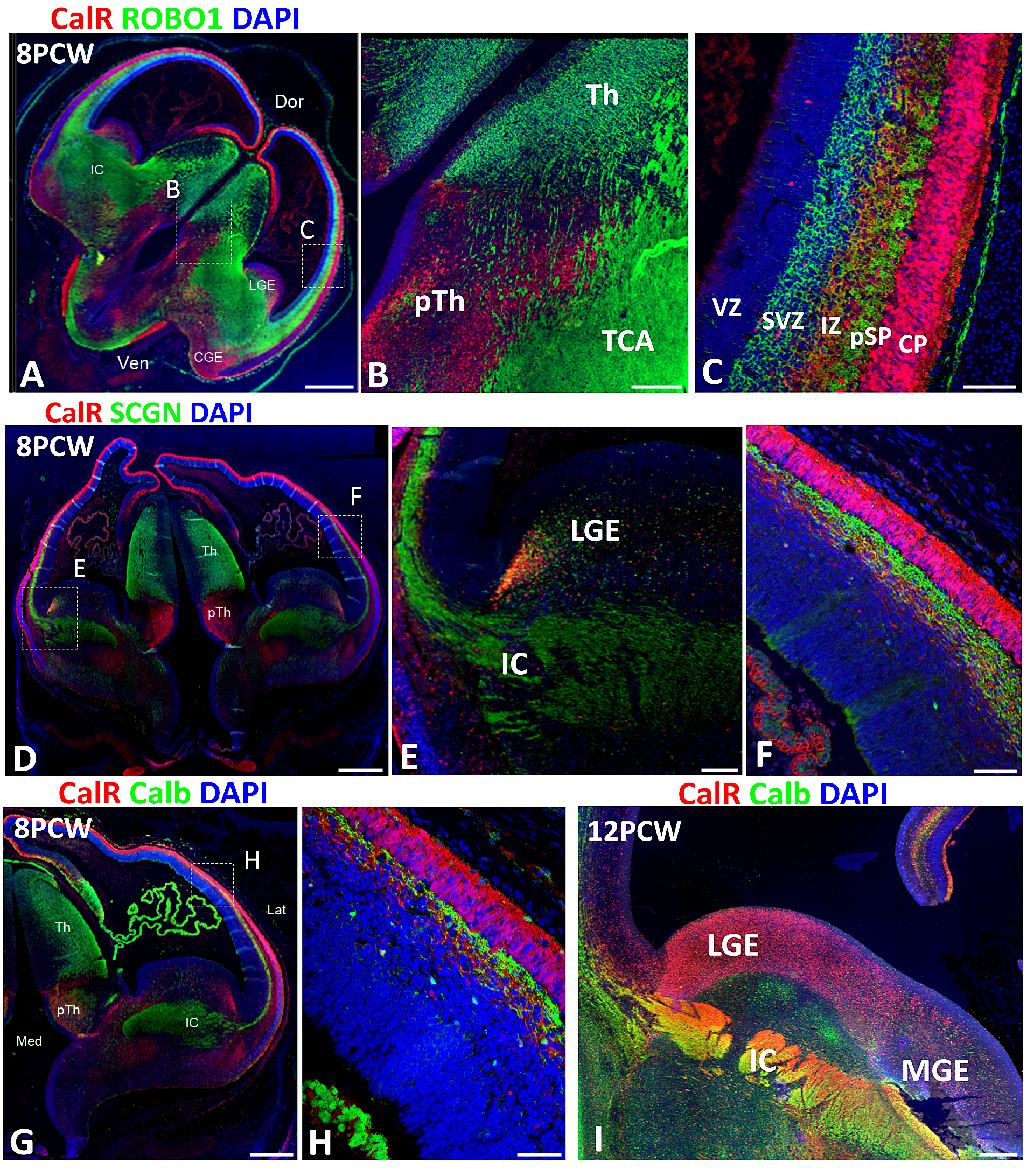

Supplement: Supplementary Data [file bhy327_supplementary_materials.zip › bhy327_Suppl_Fig_3.tif]
